# Psychometric validation of the persian short form of the physical literacy in children questionnaire in Iranian children aged 4–12 years: factorial validity, internal consistency, and gender-based measurement invariance

**DOI:** 10.1186/s12887-026-07439-5

**Published:** 2026-08-03

**Authors:** Zahra Afazeli, Behzad Mohammadi Orangi, Fahimeh Hassani

**Affiliations:** 1 Faculty of Sport Sciences, Department of Motor Behavior and Sport Management, Urmia University, Urmia, Iran; 2 Department of Sport Science, School of Humanities, Damghan University, Damghan, Iran; 3 Faculty of Sports Science, Department of Sports Science, Gedik University, Istanbul, Turkey

**Keywords:** Physical literacy, Children, Psychometric validation, Confirmatory factor analysis, Measurement invariance, Iran, PL-C Quest

## Abstract

**Objectives:**

The Physical Literacy (PL) in Children Questionnaire (PL-C Quest) is a 30-item pictorial instrument assessing children's PL across four domains. A 12-item short form has recently been developed; however, its psychometric properties have not been examined in Persian-speaking populations. This study evaluated the factorial validity, internal consistency, and measurement invariance of the Persian short form in Iranian children.

**Design:**

Pooled cross-sectional.

**Methods:**

Data from two independent validation studies conducted in Tehran were pooled, yielding a sample of 2,213 children aged 4–12 years (1,143 girls; mean age = 7.6 ± 3.51 years). Second-order confirmatory factor analysis (CFA) was performed using the WLSMV estimator. Internal consistency was evaluated using Cronbach's alpha, ordinal alpha, McDonald's omega, composite reliability, and average variance extracted (AVE). Measurement invariance across gender and age groups (4–7 vs. 8–12 years) was examined using sequential multi-group CFA.

**Results:**

The hierarchical four-factor model demonstrated excellent fit (CFI = 0.996, TLI = 0.994, RMSEA = 0.032, SRMR = 0.030). Standardized item loadings ranged from 0.570 to 0.776, while higher-order factor loadings ranged from 0.851 to 0.988. Internal split-sample cross-validation confirmed the stability of the proposed factor structure. Internal consistency was acceptable for the Physical (ω = 0.714), Cognitive (ω = 0.649), and Psychological (ω = 0.638) subscales, whereas the two-item Social subscale showed lower reliability (ω = 0.503). Composite reliability values ranged from 0.599 to 0.779 and AVE values from 0.431 to 0.471. Full scalar measurement invariance was supported across both gender (ΔCFI ≤ 0.001; ΔRMSEA ≤ 0.003) and age groups (ΔCFI ≤ 0.003; ΔRMSEA ≤ 0.005).

**Conclusions:**

The Persian short form of the PL-C Quest demonstrated a well-fitting hierarchical factor structure, acceptable internal consistency for most domains, and equivalent measurement across gender and developmental age groups. These findings support its potential utility for research and group-level assessment of PL in Iranian children. Nevertheless, evidence for convergent and discriminant validity remained limited, and important psychometric properties, including criterion validity, test–retest reliability, responsiveness, and measurement error, were not examined. Accordingly, further validation in more diverse populations and against external criteria is required before routine clinical or educational implementation can be recommended.

**Supplementary Information:**

The online version contains supplementary material available at https://doi.org/10.1186/s12887-026-07439-5.

## Introduction

Across the lifespan, sustained engagement in physical activity depends not merely on physical capacity, but on a constellation of interconnected personal attributes that together constitute what researchers increasingly term physical literacy (PL). Rather than a static trait, PL represents a dynamic developmental achievement; the integration of movement competence, psychological readiness, social awareness, and knowledge-based understanding that collectively enable an individual to participate meaningfully and voluntarily in physical activity throughout life [4]. The International PL Association frames this construct as encompassing the motivation, confidence, physical competence, and knowledge necessary for individuals to value and sustain active lifestyles [26], while national frameworks in Canada, England, and Australia have each contributed distinct yet conceptually overlapping elaborations of these core dimensions [9, 18, 24]. What unifies these diverse articulations is a shared recognition that PL is not reducible to fitness outcomes alone but constitutes a holistic developmental resource with implications extending well beyond sport participation.

The developmental significance of early childhood as a formative period for PL has gained substantial empirical support in recent years. Longitudinal evidence consistently demonstrates that fundamental movement skill proficiency, physical self-efficacy beliefs, and habitual activity patterns established during primary school years function as robust predictors of adolescent and adult physical activity behavior [2, 3]. This temporal sensitivity suggests that gaps in PL emerging during childhood are not self-correcting,rather, they tend to compound across developmental stages, creating trajectories of physical inactivity that carry well-documented consequences for metabolic, cardiovascular, and mental health outcomes [6]. From a public health perspective, identifying children who are at risk of PL deficits early, and with sufficient precision to guide targeted intervention, represents a priority that transcends academic interest and carries direct implications for educational and clinical practice.

Responding to this priority, the past two decades have witnessed a proliferation of tools designed to assess PL in children. These instruments span a broad methodological spectrum; performance-based assessments of motor skill competency and physical fitness, observational rating scales for practitioners, and self-report questionnaires targeting perceived capabilities, motivation, and knowledge [1]. Performance-based approaches, such as those incorporated in the Canadian Assessment of PL [17] or standardized motor assessments [13], offer objective measurement advantages but impose practical constraints, requiring trained assessors, specialized equipment, and dedicated space, that limit their scalability in real-world educational or community settings [16]. Self-report instruments, by contrast, offer substantially greater logistical accessibility, but their utility with younger children depends critically on whether the format adequately accommodates developmental constraints in literacy, abstract reasoning, and self-referential judgment [2, 3]. Most existing self-report tools, including the Perceived PL Inventory for Adolescents [22] and the PL Assessment for Youth tools [5], were designed for older age groups, leaving a recognized instrumentation gap for children in early-to-middle childhood.

The PL in Children Questionnaire (PL-C Quest), developed by Barnett et al. [2, 3], represents a methodologically distinctive response to this gap. By employing a pictorial format featuring a gender-neutral cartoon rabbit across thirty activity scenarios, the instrument extends self-report measurement to children as young as four years without imposing literacy demands that would otherwise render self-completion impossible for this age group. The PL-C Quest operationalizes thirty elements of the Australian PL Framework (APLF) across four domains, physical, psychological, social, and cognitive, making it one of the few available instruments to assess PL holistically rather than selectively [9]. The psychometric evidence base supporting the PL-C Quest has expanded rapidly across multiple national contexts. Studies conducted in Australia [2, 3,10], China [27], Spain [21], the United Kingdom [25], and Greece [15] have collectively provided evidence for content validity, factorial structure, response process validity, and predictive associations with physical activity. Comparative evaluations of available PL tools have consistently positioned the PL-C Quest among the most comprehensively validated instruments for primary-school-aged children [1]. The instrument has attracted substantial international interest, with research teams spanning multiple continents seeking access within two years of its initial publication.

Despite this impressive evidentiary foundation, the practical applicability of the full thirty-item version remains constrained by completion time. Nevertheless, reported administration durations across diverse implementation settings [2, 3, 8] may represent a non-trivial time investment that challenges delivery within the compressed schedules typical of physical education lessons, health screenings, or therapy consultations, such as physical education lessons, health screenings, or occupational therapy consultations. Recognizing this feasibility tension, Carl et al., undertook a systematic derivation of a twelve-item short form of the PL-C Quest, employing a multi-indicator aggregation framework incorporating nine psychometric criteria spanning content validity, item discrimination, internal consistency, criterion validity against proxy-reported physical activity, measurement fairness across sex, and cross-cultural generalizability across four countries [7]. The resulting short form demonstrated strong domain-specific correlations with its parent instrument, confirming that a substantially abbreviated version retained the essential measurement properties of the original instrument across all four domains [7]. This approach aligns with established psychometric evidence demonstrating that considerably shorter versions of measurement instruments can preserve adequate precision when item selection follows systematic empirical criteria [23].

The cross-cultural generalizability evidence incorporated into the short-form derivation process is encouraging, but it does not substitute for context-specific psychometric validation. Measurement invariance cannot be assumed across linguistic, cultural, and structural boundaries; culturally mediated variations in children's relationships with physical activity, their interpretations of pictorial scenarios, and their understanding of concepts such as confidence, strategy, or social belonging may all influence item functioning in ways that are not transparent from validation evidence gathered elsewhere [19]. Iran presents a context in which these considerations are particularly salient. With a school-age population exceeding fourteen million and a national curriculum in which physical education is universally mandated, Iran constitutes a large and policy-relevant setting for PL research [20]. At the same time, the Iranian context carries structural and cultural characteristics that distinguish it meaningfully from the populations in which the PL-C Quest has previously been validated [14, 20]. The gender-segregated organization of public primary schooling creates distinct social environments for boys and girls, with potential implications for the development of physical self-concept, perceived social belonging in sport, and normative beliefs about active participation [14, 20]. Epidemiological data indicate that Iranian children, particularly girls, exhibit levels of physical inactivity that are among the highest documented in global surveillance datasets, yet the psychosocial and cognitive determinants of this pattern remain poorly characterized due to the absence of validated measurement tools appropriate for this age group. Furthermore, Iranian children's cultural familiarity with the movement contexts depicted in PL-C Quest pictorial stimuli, originally developed within an Australian framework, cannot be taken for granted, making a rigorous cross-cultural adaptation and cognitive validation process indispensable. Notably, no existing validation study has examined whether the pictorial scenarios of the PL-C Quest, originally conceived within an Australian cultural framework, are interpreted consistently by children socialized within a Persian-speaking, Islamic educational context, where movement norms, gender dynamics in sport, and children's relationship with competitive physical activity differ substantially from Western reference populations.

Despite the growing international validation literature surrounding the PL-C Quest and its short form, no published study has examined the psychometric properties of either version in an Iranian pediatric population. This absence constitutes a substantive barrier; without evidence that the instrument measures the same constructs equivalently in Iranian children as in previously studied populations, its use in Iranian research, educational assessment, or clinical contexts lacks adequate empirical justification. The present study was designed explicitly to address this gap. Using data pooled from two independent cross-sectional validation studies encompassing 2,213 Iranian children aged four to twelve years, we examined the factorial validity, internal consistency, and gender-based measurement invariance of the Persian short-form PL-C Quest. Beyond psychometric characterization, this study makes a methodological contribution by extending the age coverage of the short-form validation below the eight-to-twelve-year range examined by Carl et al. [7], thereby covering the full developmental target population for which the PL-C Quest was originally designed. The findings are intended to provide researchers, physical educators, coaches, and health professionals in Iran with an empirically justified, time-efficient instrument capable of supporting both population-level surveillance and individualized assessment of children's PL.

## Methods

### Overall design and rationale

A pooled cross-sectional design was employed to derive and validate a short-form version of the Persian PL-c Quest in Iranian children aged 4–12 years. This study leveraged existing data from two previously conducted psychometric validation studies – one focusing on children aged 4–8 years and the other on children aged 8–12 years – which were integrated into a single comprehensive dataset spanning early and middle childhood. The short-form was derived by selecting 12 items from the full 30-item instrument based on a multi-indicator psychometric approach, consistent with the methodology of Carl et al. [7]. No new or hybrid items were introduced, thereby preserving the theoretical integrity of the original APLF. Although the original short-form was derived in children aged 8–12 years [7], the present study extends its validation downward to include children as young as 4 years, thereby covering the full target age range of the PL-c Quest.

#### Pooled sample (children aged 4–12 years)

**Fig. 1:**
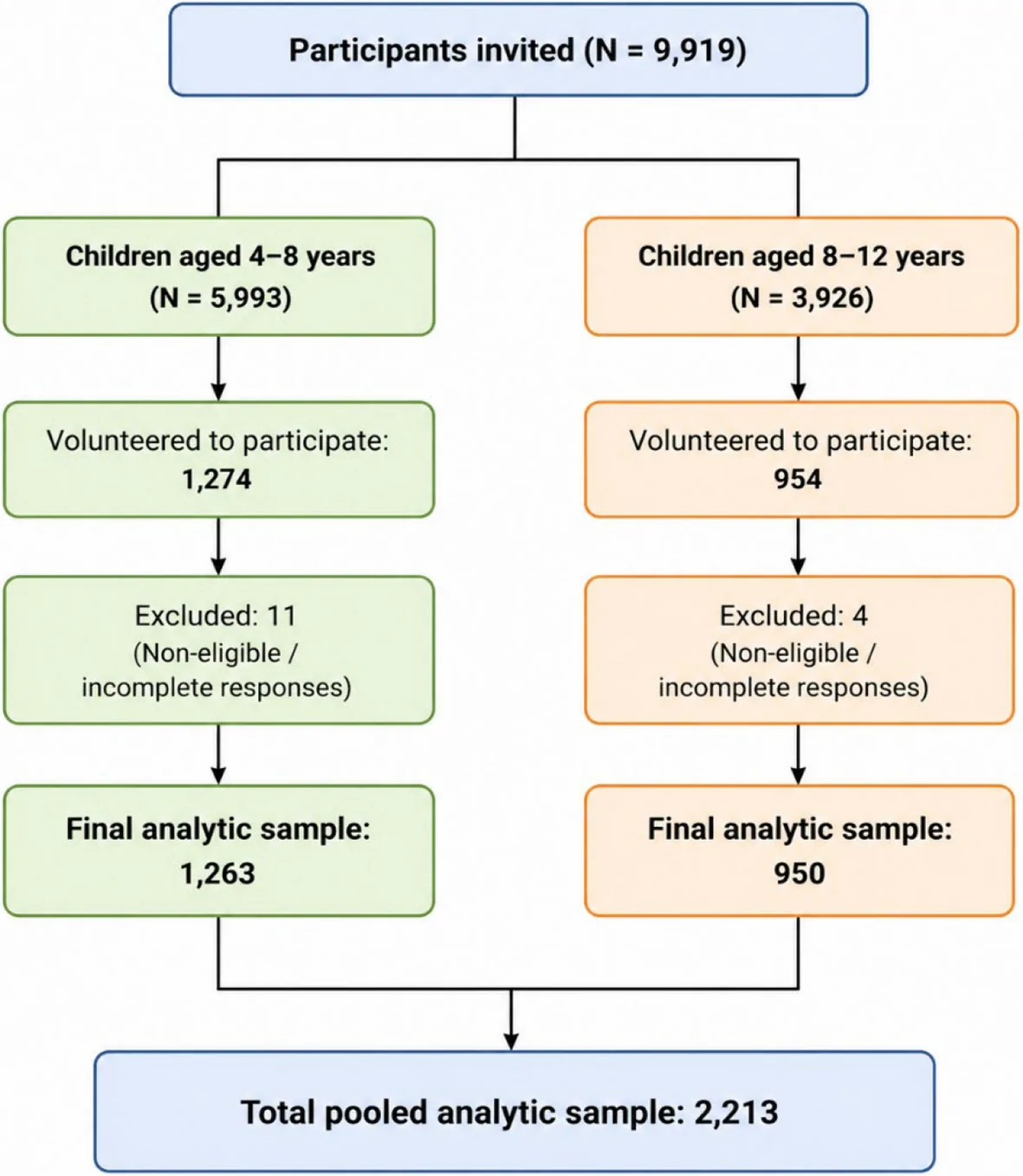
Flowchart of participant selection and inclusion in the study

The final pooled sample comprised 2213 children aged 4–12 years. The overall process of selecting participants is shown in Fig. 1. This sample size substantially exceeds that of the original Australian validation study (*N* = 620; [7] and provides excellent statistical power for multi-group invariance testing across gender and age groups. All participants had completed the full 30-item Persian PL-c Quest, and short-form domain and total scores were extracted from these responses.Sampling procedures combined purposive school selection (for the older cohort) with random cluster sampling at the classroom level within selected institutions, ensuring representation of the urban population of Tehran. Both cohorts were assessed using the same instrument, the same inclusion and exclusion criteria, and comparable recruitment procedures. The only procedural difference between cohorts was that, due to developmental considerations, children aged 4–8 years completed the instrument individually with an assessor seated beside them to ensure comprehension, whereas children aged 8–12 years self-completed the questionnaire with minimal supervision. Before pooling the datasets, distributions of key variables were examined and no meaningful cohort differences were observed. Because the instrument is designed for children aged 4–12 years but separates administration into two recommended age bands (4–8 years and 8–12 years), data collection was carried out in two phases. To obtain a sufficiently large sample and because the process was time-consuming, data for each age band were collected separately, while all administration details and inclusion and exclusion criteria were kept identical across both phases.

#### Cohort 1: younger children (4–8 years)

Data for the younger cohort were drawn from a cross-sectional validation study of the Persian PL-c Quest in children aged 4 to 8 years (including 8-year-olds only in this cohort to avoid overlap). A total of 1,263 children (mean age = 6.49 ± 1.35 years; 52.9% boys) were recruited from kindergartens, child training centers, and sports clubs across all urban districts of Tehran between August and September 2025. A multistage cluster random sampling approach was used: first, institutions were identified from comprehensive lists; second, classrooms or groups within each institution were randomly assigned numerical codes and selected using a computer-generated sequence; third, all children within selected clusters were invited to participate. This procedure ensured transparency, reproducibility, and minimization of selection bias. Cognitive interviewing and pilot testing confirmed that the pictorial format was developmentally appropriate for this age group.

#### Cohort 2: older children (8–12 years)

Schools were purposively selected to represent the gender-segregated structure of the Iranian state school system, and random cluster sampling was then applied at the classroom level within each selected school. Data for the older cohort was obtained from a cross-sectional validation study conducted in public primary schools in Tehran. A total of 950 children (mean age = 9.40 ± 0.96 years; 57.6% boys) were recruited between February and March 2025. Using the same multistage cluster random sampling method, five schools were purposively selected to represent the gender-segregated structure of the Iranian state school system (three girls’ schools, two boys’ schools), and all classes within grades 2 through 6 were invited to participate. Of 3,926 eligible students, 954 returned signed parental consent forms (initial consent rate: 24.3%). Following data quality screening, 4 questionnaires were excluded due to patterned responding (i.e., identical response option selected across all items within a domain). No item-level missing data were present in the remaining 950 questionnaires, which formed the final analytic sample for this cohort.

#### Ethical considerations

Ethical approval for both constituent studies was obtained from the Istanbul Gedik University Health Sciences Ethics Committee, Istanbul Gedik University (Approval Code: E-56365223–050.04–2024.137548.208; Decision No: 2024/10, dated October 30, 2024). The study was conducted in accordance with the principles of the Declaration of Helsinki. Written informed consent was obtained from parents or legal guardians of all participating children, and age-appropriate verbal assent was obtained from each child prior to participation. Participants were informed of their right to withdraw at any time without consequence, and confidentiality of personal information was strictly maintained.

### Instruments and measures

#### The full Persian PL-c quest (30-item parent instrument)

##### Original instrument and authorization

The original PL-c Quest was developed by Barnett et al [2, 3] within the APLF. This pictorial self-report instrument comprises 30 items distributed across four domains: physical domain (12 items), psychological/affective domain (7 items), social domain (4 items), and cognitive domain (7 items). Each item is scored using a 4-point pictorial response scale (1 = "totally unlike me" to 4 = "totally like me"). The total score ranges from 30 to 120, with higher scores indicating greater perceived PL. Formal authorization and full access to the original instrument were obtained from the APLF Association. Final approval of the adapted version was provided by Professor Lisa Barnett, the original developer of the instrument.

##### Cross-cultural adaptation (five-stage protocol)

The adaptation of the Persian version from the original English instrument followed a rigorous five-stage protocol aligned with international guidelines for cross-cultural adaptation of self-report measures (Beaton et al. 2000) and psychometric instrument development [19]. The stages of this protocol are as follows:

- Stage 1: Forward Translation. Two independent bilingual translators, one with expertise in psychometrics and the other in physical education, produced separate Persian translations of the original instrument. Translators were instructed to prioritize conceptual equivalence over literal correspondence, paying particular attention to age-appropriate language and culturally familiar movement contexts.
- Stage 2: Back-Translation. Two additional bilingual translators, both native Persian speakers with academic backgrounds in English language education and unfamiliar with the original instrument, independently backtranslated the Persian version obtained from the previous stage into English. Discrepancies between the back-translated version and the original English were reviewed to identify and resolve potential conceptual distortions introduced during forward translation.
- Stage 3: Expert Panel Review. A multidisciplinary committee consisting of two professors of physical education, one psychometrician, and one linguist reviewed all translation versions alongside the original. This panel evaluated three types of equivalence: semantic equivalence (ensuring words and phrases conveyed the same meaning), conceptual equivalence (ensuring items measured the same construct across cultures), and cultural relevance (ensuring items wereappropriate for Iranian children aged 4–12 years). Special attention was given to avoiding value-laden expressions, ensuring age-appropriate terminology, and maintaining consistency with the theoretical constructs of the APLF.
- Stage 4: Developer Consultation. The back-translated version was reviewed by Professor Lisa Barnett, the original developer of the PL-c Quest. Her expert feedback emphasized conceptual accuracy, precision of terminology, and cultural appropriateness. Several refinements were introduced to strengthen the equivalence of the Persian version with the original: terminology was adjusted to improve linguistic accuracy (e.g., "bicycling" replaced "riding a bicycle"; "exercise" was revised to "playing sport"), items in the affective domain were clarified with more concrete examples (e.g., rephrasing "miss the target"), activity descriptors were corrected (e.g., "sledding" was replaced with "zipline"), and object references were standardized (e.g., "stone" revised to "rock"). Subject terminology was harmonized by consistently using "children" instead of "kids," and value-laden expressions implying judgment (e.g., "not as good at jumping") were removed to preserve neutrality and age-appropriateness.
- Stage 5: Final Approval and Documentation. This iterative process of translation, reconciliation, back-translation, expert review, and developer feedback ensured that the Persian version of the PL-c Quest achieved semantic, conceptual, and functional equivalence. Importantly, no changes were made to the pictorial representations or scoring system, thereby preserving the integrity of the original instrument while ensuring its applicability in the Persian context.

##### Pilot testing and cognitive interviewing

Following the adaptation stages, the consensus Persian version was administered separately to purposive samples of children in the target age groups (4–8 years and 8–12 years). For the 4–8 year age group, a sample of 20 children (mean age = 6.26 years) underwent cognitive interviewing using think-aloud and standardized probing methods (Willis & Miller 2011). For the 8–12 year age group, 20 children were similarly assessed under the same protocol. The cognitive interviews evaluated four response processes: comprehension (whether children understood the item wording and pictorial content as intended), retrieval (whether children could access relevant movement experiences), judgment (whether children could assess the applicability of scenarios to themselves), and response mapping (whether children could accurately translate their judgment onto the 4-point scale). Results indicated that the pictorial stimuli were interpreted consistently across participants; minor adjustments were made to the wording of some items, and two pictorial icons were modified to better reflect Iranian movement contexts. Subsequently, a pilot administration was conducted with 30 children in each age group, confirming satisfactory response rates and the absence of floor or ceiling effects at the item level. The final Persian version retained all 30 original items in their four-domain structure. To provide more detail on the cognitive interviewing process, two rounds of interviews were conducted for each age group. In the first round, children were asked to explain which picture they identified with (“very similar to me” or “a little similar to me”) and why. This process helped identify items where younger children had difficulty interpreting specific terms (such as “coordination” or “confidence”) or where the contrast between the two pictorial options was not sufficiently clear. In the second round, revised wording and clearer pictorial contrasts were tested. Examples included simplifying action verbs, clarifying emotional terms, and adjusting two icons to better reflect familiar movement contexts for Iranian children. All interview notes were coded using a structured coding approach, including categories such as lexical clarity, pictorial clarity, developmental appropriateness, and response mapping. Revisions were made only when at least two coders independently agreed that an item required modification.

#### The Persian short-form (PL-c quest)

##### Selection of items based on Carl et al. [7]

The item selection for the short version followed the multi-indicator framework proposed by Carl et al. [7]. In that framework, nine psychometric indicators are aggregated across six domains of measurement quality: content validity (standardised factor loading), item discrimination (item difficulty and corrected item-total correlation), internal reliability (change in Cronbach’s α if the item is deleted), criterion validity (univariate and multivariate correlations with proxy-reported moderate-to-vigorous physical activity), test fairness (separate eligibility scores for boys and girls), and cross-cultural generalisability (factor loadings from validation studies in China, Spain, the United Kingdom, and Greece). Although the original framework included criterion validity based on MVPA, this aspect could not be examined in the present study due to the retrospective nature of the pooled dataset (see Limitations). Nevertheless, the remaining eight indicators supported the selection of the 12 items, and the strong cross-cultural generalisability scores reported for these items across multiple countries (China, Spain, United Kingdom, Greece) justified adopting the identical item set for the Persian context.

To preserve the proportional domain structure of the original 30-item instrument, the short version was designed to include four items from the physical domain (out of 12), three from the psychological domain (out of seven), two from the social domain (out of four), and three from the cognitive domain (out of seven). For the physical domain, an a-priori rule required at least one movement-skill item in addition to fitness-related items, in line with the recommendation of Dudley and Cairney [11] to avoid an overemphasis on fitness.

##### Final structure of the Persian short‑form (PL-c Quest)

The Persian version of the short form of the PL-C Quest retained the four-domain structure of the original instrument and comprised twelve items across the physical, psychological, social, and cognitive domains (Supplementary File 1). The physical domain contained four items, namely movement skills (using a jumping-over-obstacles scenario as a fundamental movement skill), cardiovascular endurance (long-distance running), muscular endurance (performing repeated strength movements), and speed (short-distance fast running). The psychological domain included three items measuring motivation (willingness to initiate physical activity), confidence (certainty about one’s own movement abilities), and engagement and enjoyment (feeling of happiness during physical activity). The social domain consisted of two items assessing, respectively, relationships (positive peer interaction in a movement setting) and society and culture (understanding social norms in physical activity). Finally, the cognitive domain comprised three items covering content knowledge (awareness of the rules and principles of movement activities), strategy and planning (designing a movement plan to achieve a goal), and tactics (quick decision-making in competitive movement situations).

For each participant in the pooled Iranian sample (*N* = 2213), domain and total short-form scores were computed directly from their responses to the full 30-item questionnaire. In the original derivation study [7], the short-form domain sum scores correlated strongly with the corresponding full-version domain scores (range 0.74 ≤ r ≤ 0.84), indicating that the short-form retains sufficient information from the original instrument.

To confirm the factorial validity of the Persian short-form, a confirmatory factor analysis (CFA) was conducted on the pooled Iranian sample. Acceptable model fit was defined as CFI/TLI ≥ 0.90, RMSEA ≤ 0.08, and SRMR ≤ 0.08. The four-domain correlated-traits model, specified according to the original four-domain factor structure, showed acceptable to good fit (CFI = 0.920, TLI = 0.905, RMSEA = 0.059, SRMR = 0.043). These fit indices closely parallel those reported by Carl et al. [7] for the Australian sample, supporting the transferability of the short-form structure to the Persian context.

### Data collection procedures

For the present study, data were drawn from two independent prior validation studies, each following a data collection protocol tailored to its target age group. The administration procedures in both studies were aligned with the pictorial nature of the instrument and the developmental level of the children. Below, the administration protocols for each age group, the training of research assistants, and the data quality control and screening methods are described.

#### Administration protocol for younger children (4–8 years)

In the study involving children aged 4–8 years, data were collected through individual, face-to-face administration in child-centered environments, including kindergartens, child training centers, and sports clubs. Each child was assessed separately, with a trained evaluator seated beside the participant to ensure comprehension of the items and accurate recording of responses. Participation was voluntary and took place only when the child was ready and willing to respond, thus minimizing potential stress and ensuring authentic engagement. The evaluation was conducted sequentially: after one child completed the questionnaire, the next child who expressed readiness and interest was invited to participate. Although this individualized, one-on-one procedure reduced the number of assessments completed per day, it was considered essential for the 4–8-year age group, as children at this developmental stage require greater attention, patience, and support to comprehend the items and provide meaningful responses. Experience suggests that such a time-intensive approach enhances administration accuracy and, consequently, the reliability and validity of the data collected. During administration, the pictorial version of the PL-c Quest was used. For younger children, the evaluator read the accompanying statements aloud and guided the child through a two-step response process: first asking which of the two images was more like them, and then whether that image was "very much like me" or "a little like me." This standard procedure, derived from the user guide of the original instrument, has also been used in previous validation studies in China and the United Kingdom.

#### Administration protocol for older children (8–12 years)

In the study involving children aged 8–12 years, data collection was conducted between February 1 and March 11, 2025, in public primary schools in Tehran. Trained research assistants administered the questionnaire individually in classrooms using a standardised administration script. Pictorial prompts were presented to children one at a time, and the response options were explained using the accompanying pictorial scale before the start. Assessors were instructed to read the items aloud, display the pictorial stimuli clearly, and refrain from coaching or influencing the children's responses. Children completed the questionnaire in a single session lasting approximately 15–20 min.

The main difference in administration for this age group was that children aged 8–12 years were able to self‑complete the questionnaire with minimal guidance, while the assessors monitored the process to ensure accurate completion. Furthermore, unlike the younger age group, group administration (several children simultaneously in one classroom) was possible for this age range, although emphasis was placed on individual completion to maintain focus and reduce errors. This protocol is consistent with the methods reported in international validation studies conducted in China, Spain, and the United Kingdom.

#### Training research assistants

In both studies, prior to data collection, all research assistants participated in a standardised training programme. This training included detailed instructions on the administration protocol, full familiarisation with the questionnaire items (including both pictorial and text versions), and practice sessions with pilot participants. The practice sessions allowed the assessors to rehearse the procedures in realistic conditions, identify potential challenges (such as how to handle children who became confused or were reluctant to cooperate), and refine their communication strategies with children. Through these preparatory activities, the evaluators developed a shared understanding of the assessment process, which minimised between‑site variability and enhanced the reliability of the data collection. To reduce possible bias, the assessors were strictly instructed not to prompt or influence the children's choices in any way, and responses were recorded immediately to avoid recall errors. Aligning the protocols with those used in previous studies in China and the United Kingdom strengthened methodological comparability across different cultural contexts.

#### Data quality control and screening

After data collection was completed in both age groups, quality control and screening process was carried out in several stages:

- Stage 1: Checking for Patterned Responding. Questionnaires in which the participant had selected an identical response option across all items of a particular domain (e.g., all responses "totally like me") were considered to exhibit patterned responding. Such cases were excluded from the final analysis, as they indicate a lack of meaningful engagement with the item content. In the 8–12-year group, 4 questionnaires were excluded for this reason. In the 4–8-year group, questionnaires with identical response patterns on more than 80% of items were similarly reviewed and excluded; however, no such cases were identified in this cohort.
- Stage 2: Missing Data Assessment. In both age groups, after the exclusion of patterned responders, there were no items missing data. This was likely due to the face‑to‑face administration and the direct supervision of the assessors, which prevented missed responses. Consequently, no imputation procedures were required in either study.
- Stage 3: Floor and Ceiling Effects. For each item, the distribution of responses was examined to determine whether more than 80% of the responses were concentrated in the lowest (1) or highest (4) option. In the pilot administration (30 children per age group), no item showed appreciable floor or ceiling effects. In the final analysis, the proportion of extreme responses did not exceed the critical threshold for any item.
- Stage 4: Consent Rate and Reasons for Non‑participation. In the 8–12-year group, the parental consent rate was 24.3% (954 out of 3,926). The main reasons for non‑participation included the return of unsigned consent forms, child unwillingness, and scheduling constraints. Although a low consent rate can raise the risk of selection bias, the final sample size (*n* = 950) and the gender and socioeconomic diversity of the selected schools partly mitigate this concern. In the 4–8-year group, because sampling was carried out through cooperati­ng centres (kindergartens and clubs), the participation rate was substantially higher, and missing data due to momentary child refusal were minimal.
- Stage 5: Data Registration and Storage. All data were stored digitally on a secure, password‑protected server (Porsline, https://porsline.ir/) located in Iran, compliant with local data protection regulations. Access was restricted to the research team.

### Statistical analysis

All statistical analyses were conducted using IBM SPSS Statistics version 26 (IBM Corp., Armonk, NY, USA) and R version 4.5.1 (R Foundation for Statistical Computing, Vienna, Austria). Confirmatory factor analyses and measurement invariance testing were performed using the lavaan package (version 0.6–19). None of the questionnaire items required reverse coding; all items were scored in the same direction, with higher scores indicating higher levels of PL. Descriptive statistics were calculated for all study variables. Floor and ceiling effects were evaluated for the total sample by calculating the percentage of participants obtaining the lowest and highest possible scores, respectively; values below 15% were considered acceptable. Factorial validity was examined using CFA with the weighted least squares mean and variance adjusted (WLSMV) estimator, which is appropriate for ordered categorical data. Two competing models were evaluated: a correlated four-factor first-order model and the hypothesized hierarchical second-order model in which the four domains (Physical, Psychological, Social, and Cognitive) loaded onto a higher-order PL factor. Model fit was assessed using the chi-square statistic (χ^2^), Comparative Fit Index (CFI), Tucker–Lewis Index (TLI), Root Mean Square Error of Approximation (RMSEA) with its 90% confidence interval, and Standardized Root Mean Square Residual (SRMR). Model fit was considered acceptable when CFI and TLI were ≥ 0.95, RMSEA was ≤ 0.06, and SRMR was ≤ 0.08. Measurement invariance was examined across gender (girls vs. boys) and age groups (4–8 years vs. 8–12 years) using multigroup CFA. Configural, metric, and scalar invariance were evaluated sequentially. Invariance was supported when changes in comparative fit indices met the recommended criteria of ΔCFI ≤ 0.010 and ΔRMSEA ≤ 0.015. Internal consistency was assessed using Cronbach's alpha, ordinal alpha, McDonald's omega, composite reliability (CR), and average variance extracted (AVE). Composite reliability values ≥ 0.70 were considered acceptable, whereas AVE values were interpreted alongside standardized factor loadings and composite reliability rather than as a standalone criterion. Discriminant validity was examined using both the Fornell–Larcker criterion and the heterotrait–monotrait ratio (HTMT). To further evaluate the stability of the measurement model, an internal split-sample cross-validation was conducted by randomly dividing the total sample into two approximately equal subsamples and fitting the second-order CFA model separately in each subsample.

## Results

### Results

#### Participants and descriptive statistics

A total of 2,213 children participated in the present study, comprising 1,143 girls and 1,070 boys. For the full sample, mean body weight was 27.7 kg (SD = 9.38), mean height was 122.5 cm (SD = 15.40), and mean age was 7.6 years (SD = 3.51). Among girls, mean body weight was 28.60 kg (SD = 10.32), mean height was 124.20 cm (SD = 15.06), and mean age was 7.78 years (SD = 3.49). Among boys, mean body weight was 26.80 kg (SD = 8.16), mean height was 120.68 cm (SD = 15.58), and mean age was 7.32 years (SD = 3.52). Regarding PL scores, the full sample demonstrated a mean Total PL score of 36.5 (SD = 7.32), with subscale means of 12.0 (SD = 2.94) for Physical, 9.3 (SD = 2.25) for Psychological, 6.2 (SD = 1.61) for Social, and 9.0 (SD = 2.26) for Cognitive. Girls demonstrated a mean Total PL score of 36.87 (SD = 7.23), with subscale means of 12.09 (SD = 2.90) for Physical, 9.32 (SD = 2.24) for Psychological, 6.30 (SD = 1.59) for Social, and 9.17 (SD = 2.24) for Cognitive. Boys demonstrated a mean Total PL score of 36.18 (SD = 7.42), with subscale means of 11.88 (SD = 2.98) for Physical, 9.22 (SD = 2.26) for Psychological, 6.16 (SD = 1.65) for Social, and 8.92 (SD = 2.28) for Cognitive.

### Confirmatory factor analysis

To examine the factorial validity of the questionnaire, a second-order CFA was conducted using the robust weighted least squares estimator (WLSMV), which is appropriate for ordinal data. The hypothesized model consisted of four first-order factors Physical (Q1–Q4), Psychological (Q5–Q7), Social (Q8–Q9), and Cognitive (Q10–Q12) loading onto a higher-order general factor representing Total PL. Model fit was evaluated using the following criteria: CFI and TLI ≥ 0.95, RMSEA ≤ 0.06, and SRMR ≤ 0.08. The hypothesized second-order model demonstrated excellent fit to the data (χ^2^(50) = 162.328, CFI = 0.996, TLI = 0.994, RMSEA = 0.032, 90% CI = 0.026–0.037, SRMR = 0.030), indicating that the proposed hierarchical structure adequately represented the observed data.

For comparison purposes, a correlated first-order CFA model was also estimated using the same item-factor structure, WLSMV estimator, and model specification, without correlated residuals or post-hoc model modifications. Although modification indices were examined, no theoretically meaningful correlated residuals were added because none were considered sufficiently justified, and the hypothesized measurement model was retained without post-hoc modifications.The correlated first-order model demonstrated slightly better statistical fit (χ^2^(48) = 122.839, CFI = 0.997, TLI = 0.996, RMSEA = 0.027, SRMR = 0.026) than the second-order hierarchical model. Nevertheless, because the second-order model was specified a priori based on the theoretical conceptualization of PL and permits estimation of an overall PL construct, it was retained for all subsequent analyses.

**Fig. 2:**
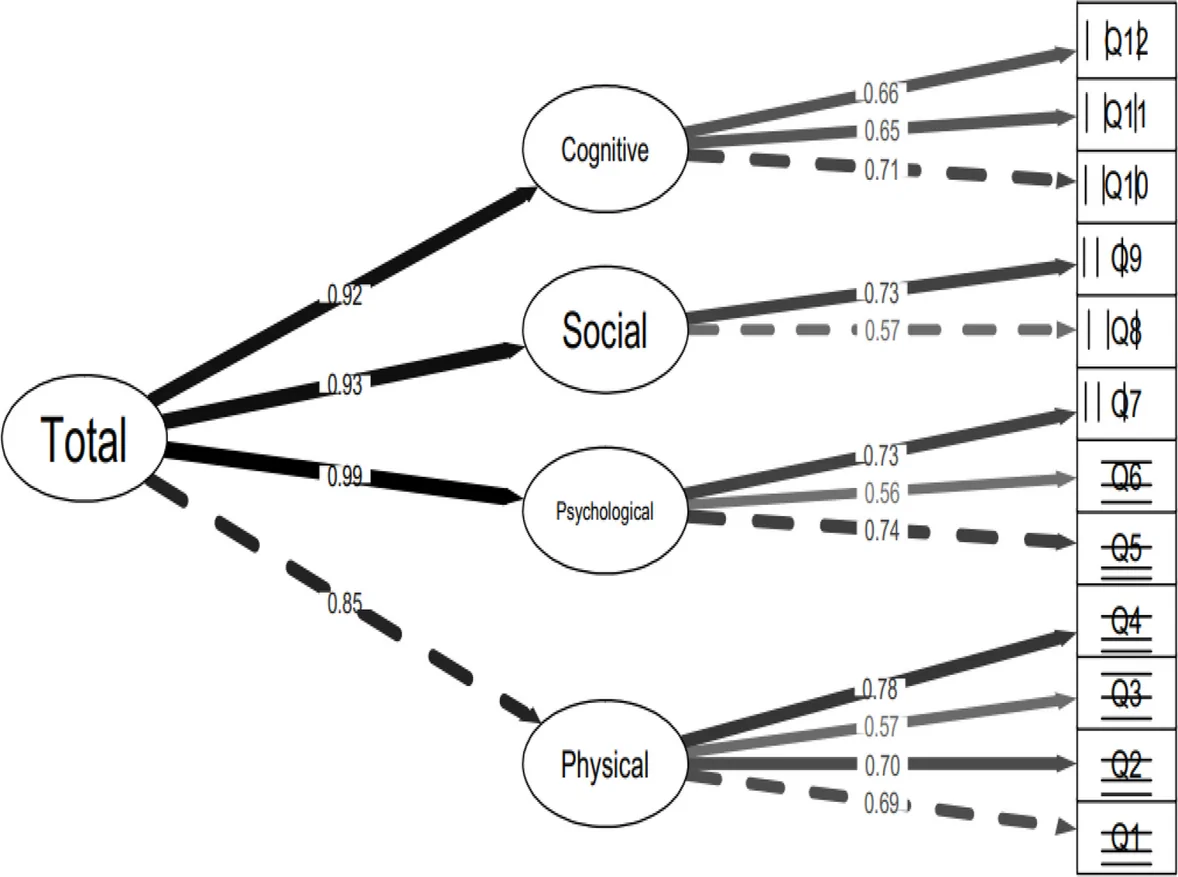
Standardized path diagram of the four-factor hierarchical CFA model with a second-order total factor. *Note.* All loadings are standardized. The figure displays item-level loadings on first-order latent factors (Physical, Psychological, Social, Cognitive) and the loadings of first-order factors onto the higher-order Total construct. All paths were statistically significant (*p* <.001)

All first-order factor loadings were statistically significant (*p* < 0.001). Standardized item loadings ranged from 0.570 to 0.776, indicating substantial relationships between observed variables and their respective latent constructs. The strongest loadings were observed within the Physical factor (0.570–0.776) and the Cognitive factor (0.654–0.714). Psychological items were loaded between 0.556 and 0.744, and social items ranged from 0.574 to 0.730. The loadings of the four first-order factors onto the second-order Total factor were all statistically significant (*p* < 0.001). Standardized loadings ranged from 0.851 (Physical) to 0.988 (Psychological), supporting a meaningful hierarchical structure (see Table 1). Psychological showed the strongest contribution to the higher-order construct (0.988), followed by Social (0.925), Cognitive (0.921), and Physical (0.851). The standardized path diagram of the final model is presented in Fig. 2.

**Table 1:** Standardized loadings of first-order factors on the second-order total factor

**Factor**	**Items**	**Std. Loading on Total**	*p*
Physical	Q1–Q4	0.851	<.001
Psychological	Q5–Q7	0.988	<.001
Social	Q8–Q9	0.925	<.001
Cognitive	Q10–Q12	0.921	<.001

*Note.* All loadings were statistically significant at *p* <.001. Estimation method: WLSMV

A second-order CFA model was evaluated because the PL-C Quest is conceptually based on PL as an overarching construct reflected by four related domains. To enable a direct comparison, both the correlated first-order and second-order models were estimated under identical specifications using the WLSMV estimator, without correlated residuals or post-hoc model modifications. As expected, the correlated first-order model demonstrated slightly better fit than the second-order model, reflecting the additional constraints imposed by the hierarchical structure. Nevertheless, the second-order model also demonstrated good overall fit, supporting its use as a theoretically meaningful representation of an overall PL construct. To further examine the stability and generalizability of the proposed measurement model, an internal split-sample cross-validation was performed. The total sample was randomly divided into two approximately equal subsamples, and the second-order CFA was independently estimated in each subsample using the same model specification and WLSMV estimator. Both subsamples demonstrated excellent model fit (Subsample 1: CFI = 0.998, TLI = 0.997, RMSEA = 0.023, SRMR = 0.029; Subsample 2: CFI = 0.994, TLI = 0.993, RMSEA = 0.034, SRMR = 0.037). Furthermore, the standardized factor loadings were highly comparable across the two subsamples, indicating that the factor structure was stable and replicable within the study sample. These findings provide additional support for the robustness of the proposed second-order measurement model.

### Measurement invariance

Measurement invariance across gender (girls vs. boys) and age groups (4–8 years vs. 8–12 years) was examined using multi-group CFA with the WLSMV estimator. The hierarchical four-factor model with a second-order Total factor was tested sequentially at the configural, metric, and scalar levels. Changes in CFI (ΔCFI) and RMSEA (ΔRMSEA) were used as the primary criteria for evaluating invariance, with |ΔCFI|≤ 0.010 and |ΔRMSEA|≤ 0.015 indicating acceptable invariance.

### Measurement invariance across gender

Configural Invariance. The configural model, which imposed no equality constraints across groups, demonstrated excellent fit: χ^2^(100) = 199.557, CFI = 0.996, TLI = 0.995, RMSEA = 0.030, and SRMR = 0.033. These results confirm that the four-factor hierarchical structure holds equivalently across girl and boy groups, establishing a baseline for further invariance testing.

Metric Invariance. Metric invariance was tested by constraining factor loadings to be equal across groups. The metric model demonstrated excellent fit: χ^2^(111) = 209.799, CFI = 0.996, TLI = 0.995, RMSEA = 0.028, and SRMR = 0.034. The change in fit relative to the configural model was ΔCFI = + 0.000 and ΔRMSEA = − 0.002, both well within recommended guidelines. Metric invariance was therefore supported, indicating that factor loadings are equivalent across gender.

Scalar Invariance. Scalar invariance was examined by additionally constraining item thresholds to be equal across groups, as appropriate for ordinal indicators. The scalar model showed excellent fit: χ^2^(123) = 209.324, CFI = 0.997, TLI = 0.996, RMSEA = 0.025, and SRMR = 0.033. The change relative to the metric model was negligible (ΔCFI = + 0.001; ΔRMSEA = − 0.003), well within recommended guidelines. Full scalar invariance across gender was therefore supported, confirming that meaningful latent mean comparisons between girl and boy groups are justified.

### Measurement invariance across age groups

Configural Invariance. The configural model, which imposed no equality constraints across age groups, demonstrated excellent fit: χ^2^(100) = 202.206, CFI = 0.996, TLI = 0.994, RMSEA = 0.030, and SRMR = 0.035. These results confirm that the four-factor hierarchical structure holds equivalently across children aged 4–8 years and 8–12 years, establishing a baseline for subsequent invariance testing.

Metric Invariance. Metric invariance was tested by constraining factor loadings to be equal across age groups. The metric model demonstrated excellent fit: χ^2^(111) = 264.239, CFI = 0.993, TLI = 0.992, RMSEA = 0.035, and SRMR = 0.039. The change in fit relative to the configural model was ΔCFI = − 0.003 and ΔRMSEA = + 0.005, both remaining well within the recommended criteria for measurement invariance. Metric invariance was therefore supported, indicating that factor loadings are equivalent across the two developmental age groups.

Scalar Invariance. Scalar invariance was examined by additionally constraining item thresholds to be equal across age groups, as appropriate for ordinal indicators. The scalar model demonstrated excellent fit: χ^2^(123) = 249.585, CFI = 0.995, TLI = 0.994, RMSEA = 0.031, and SRMR = 0.036. Relative to the metric model, only negligible changes were observed (ΔCFI = + 0.002; ΔRMSEA = − 0.004), remaining well below the recommended cutoffs. Full scalar invariance across age groups was therefore supported, confirming that meaningful latent mean comparisons between children aged 4–8 years and 8–12 years are justified.

### Internal consistency

Internal consistency of each subscale was evaluated using Cronbach's alpha, ordinal alpha, and McDonald's omega coefficients, along with AVE. Ordinal alpha was reported in addition to standard alpha given the ordinal nature of the response scale. Results are summarized in Table 2. The Physical subscale demonstrated the highest reliability (α = 0.706, α_ord = 0.771, ω = 0.714), followed by the Cognitive subscale (α = 0.648, α_ord = 0.716, ω = 0.649). The Psychological subscale showed acceptable reliability (α = 0.627, α_ord = 0.710, ω = 0.638), while the social subscale yielded lower but noteworthy values (α = 0.501, α_ord = 0.591, ω = 0.503). Across all subscales, ordinal alpha values were consistently higher than standard alpha, reflecting the ordinal nature of the items. AVE values ranged from 0.431 (Social) to 0.471 (Physical) and mean standardized factor loadings ranged from 0.652 (Social) to 0.683 (Physical), confirming adequate coherence of items within each factor. Results are presented in Table 3.

**Table 2:** Internal consistency indices for each subscale

**Factor**	**Items**	**Mean Loading**	α (95% CI)	**α (ordinal)**	ω (95% CI)	AVE (95% CI)
Physical	Q1–Q4	0.683	0.706 (0.686–0.731)	0.771	0.714 (0.690–0.738)	0.471 (0.451–0.491)
Psychological	Q5–Q7	0.676	0.627 (0.600–0.659)	0.710	0.638 (0.614–0.662)	0.464 (0.443–0.485)
Social	Q8–Q9	0.652	0.501 (0.460–0.540)	0.591	0.503 (0.480–0.526)	0.431 (0.411–0.451)
Cognitive	Q10–Q12	0.676	0.648 (0.618–0.677)	0.716	0.649 (0.625–0.673)	0.458 (0.438–0.478)

*Note.* Mean Loading = mean standardized factor loading from the CFA solution. α = Cronbach's alpha; α (ordinal) = ordinal alpha; ω = McDonald's omega; AVE = Average Variance Extracted. *N* = 2,213. All factor loadings were statistically significant at *p* <.001

**Table 3:** Composite reliability and average variance extracted

Factor	CR	AVE
Physical	0.779	0.471
Psychological	0.719	0.464
Social	0.599	0.431
Cognitive	0.717	0.458

Composite Reliability values ranged from 0.599 to 0.779 across the four latent domains. AVE values ranged from 0.431 to 0.471 across the four latent domains.

HTMT ratios ranged from 0.783 to 0.914 across the latent variables (Table 4). The highest HTMT values were observed between Psychological–Social (0.914) and Psychological–Cognitive (0.909), while the lowest ratio was found between Physical and Cognitive (0.783).

**Table 4:** HTMT ratios between latent variables

Domains	HTMT
Physical–Psychological	0.840
Physical–Social	0.787
Physical–Cognitive	0.783
Psychological–Social	0.914
Psychological–Cognitive	0.909
Social–Cognitive	0.852

The square root of AVE and the highest inter-factor correlation for each latent variable are presented in Table 5. For all constructs, the √AVE values were lower than the corresponding highest correlations, suggesting that discriminant validity may not be fully established based on the Fornell–Larcker criterion.

**Table 5:** Fornell–Larcker criterion

Factor	√AVE	Highest Correlation
Physical	0.686	0.840
Psychological	0.681	0.914
Social	0.657	0.914
Cognitive	0.677	0.909

Additional construct validity indices, including Composite Reliability, HTMT ratios, and the Fornell–Larcker criterion, are summarized in Tables 3, 4 and 5.

Correlations among the latent variables are presented in Table 6. The strongest correlations were found between Psychological and Social (0.914) and between Psychological and Cognitive (0.909), whereas the Physical construct showed relatively lower correlations with other domains, ranging from 0.783 to 0.840.

**Table 6:** Correlations among latent variables

Factor	Physical	Psychological	Social	Cognitive
Physical	1.000	0.840	0.787	0.783
Psychological	0.840	1.000	0.914	0.909
Social	0.787	0.914	1.000	0.852
Cognitive	0.783	0.909	0.852	1.000

## Discussion

This study examined the psychometric properties of the Persian 12-item short form of the PL-C Quest in a large sample of Iranian children aged 4–12 years. The findings provide evidence supporting the factorial validity, internal consistency, and measurement invariance of the instrument across gender and age groups. Nevertheless, these results represent only a partial psychometric validation of the Persian short form. Several important measurement properties recommended by the COSMIN framework, including test–retest reliability, criterion validity, responsiveness, and measurement error, were not evaluated. Furthermore, although convergent and discriminant validity were explored using AVE, composite reliability, the HTMT ratio, and the Fornell–Larcker criterion, the evidence remained limited. Specifically, all AVE values were below the conventional threshold of 0.50, the Fornell–Larcker criterion was not fully satisfied, and some HTMT values approached or slightly exceeded recommended cutoffs. Accordingly, the evidence for construct validity should be interpreted cautiously, and additional studies incorporating external validation measures and broader psychometric evaluation are needed before the Persian short form can be considered comprehensively validated.

Probably the main contribution here is the structural validity of the Persian short form. The proposed hierarchical model with four related first-order factors feeding into a higher-order PL construct showed fit indices above standard cutoffs, suggesting that the four-domain structure of the PL-C Quest works reasonably well in an Iranian context. This matches what [7] found in the original Australian sample, as well as later validation studies in Chinese [27], Spanish [21], British [25], and Greek [15] populations using the full thirty-item version. The fact that this structure holds across such different cultural settings does hint that the four-domain model at least as measured by the PL-C Quest's picture-based format may have some cross-cultural stability, including in Persian.

The higher-order loadings showed that the Psychological subscale had the strongest link to the overall PL factor, followed by Social and Cognitive, while Physical had the weakest though still meaningful loading. This could mean that, in the Iranian context, children's feelings and motivation around movement are particularly central to their overall PL, perhaps because gender-segregated school settings shape how they access and enjoy physical activity. But it's also possible that this pattern is partly due to the Psychological subscale having more similar items, which might inflate its loading through shared method effects. The high second-order loading of the Psychological domain (β = 0.988) also warrants cautious interpretation. Although this finding suggests that psychological attributes are highly central to the higher-order PL construct in the present sample, loadings approaching unity may also indicate substantial overlap between constructs or limited discriminant validity. This interpretation is consistent with the relatively high inter-factor correlations and HTMT values observed between the Psychological domain and other domains. Therefore, the current findings should not be interpreted as evidence that the Psychological domain is interchangeable with overall PL; rather, they highlight the need for future studies employing external validation measures and alternative modeling approaches to further examine the distinctiveness of the four domains. The particularly strong correlations between the Psychological domain and both the Social and Cognitive domains further indicate that these constructs are highly interconnected. Although this pattern is compatible with the conceptualization of PL as a higher-order construct, it also underscores the importance of continuing to examine discriminant validity in future studies.

One of the more practically important findings was that the Persian short form achieved full scalar measurement invariance across girls and boys. This means the factor structure, item loadings, and item thresholds all work similarly for both groups, so any differences in latent means between them would reflect real differences rather than measurement bias. This is noteworthy because in Iran, boys and girls have separate physical education classes, and social expectations around sport can differ quite a bit by gender, so equivalence across groups wasn't a given. The fact that the pictorial format of the PL-C Quest seems to function similarly for both groups suggests it may be neutral enough to cut across some of the gender-related socialization patterns in Iranian schools.

Measurement invariance analyses also supported the applicability of the Persian short form across younger (4–8 years) and older (8–12 years) children. The configural, metric, and scalar invariance models all met the recommended criteria, indicating that the questionnaire measures the same underlying construct across these developmental stages and that item responses have comparable meanings in both age groups. This finding is particularly relevant given the broad age range covered by the present study and suggests that observed differences in PL scores between younger and older children are more likely to reflect developmental differences rather than measurement artifacts. Nevertheless, because PL develops rapidly during childhood, future longitudinal studies are needed to confirm that the instrument remains stable over time and is sensitive to developmental change.

The internal consistency results for the four subscales were somewhat mixed. The Physical subscale showed the strongest reliability, and the Cognitive subscale also reached acceptable levels for research use. The Psychological subscale gave more modest estimates, while the Social subscale fell below conventional cutoffs though the authors attribute this mainly to the fact that it has only two items, rather than to a lack of coherence in the construct itself. A similar pattern has been seen in other PL studies [15, 25, 27], since the social domain has often proven harder to measure reliably than the more clearly bounded physical and cognitive domains. It's also worth noting that ordinal alpha values were consistently higher than standard Cronbach's alpha across all subscales, which fits with the ordinal response format and suggests that the usual alpha may underestimate true reliability for these items. Compared with the original Australian short-form study by [7], which also found modest reliability for the social domain, the current findings suggest this may be more about the two-item structure than about anything specific to the Persian context. Nevertheless, two-item factors are inherently limited in their ability to achieve high internal consistency, and reliability estimates for such subscales should be interpreted with caution. As a result, scores on the Social subscale may be less precise than those of the other domains and are likely to be more appropriate for research and group-level comparisons than for individual-level interpretation or decision-making.

Although the AVE values for the subscales were slightly below the conventional threshold of 0.50, this finding should be interpreted alongside the factor loadings and composite reliability estimates rather than in isolation. As noted by Fornell and Larcker [12], convergent validity may still be considered acceptable when AVE is below 0.50 provided that composite reliability exceeds 0.60, indicating that the construct explains a substantial proportion of variance despite relatively modest AVE values. In the present study, the satisfactory standardized factor loadings and acceptable composite reliability estimates suggest that the observed AVE values likely reflect the brevity of the short-form subscales rather than poor construct representation. Nevertheless, the relatively low AVE values indicate that convergent validity should be interpreted cautiously and warrants further examination in future studies. Furthermore, discriminant validity was supported based on the HTMT criterion (or the Fornell–Larcker criterion), providing additional evidence for the distinctiveness of the measured constructs.

### Strengths and limitations

Several methodological strengths of the present study deserve acknowledgment. The pooled sample of over two thousand children represents, to the best of the authors' knowledge, the largest single dataset on which the psychometric properties of the PL-C Quest short form have been examined to date, lending the structural and invariance analyses a degree of statistical power that exceeds that of most prior validation studies in this area. Furthermore, the deliberate integration of two independently conducted cohorts spanning the full four-to-twelve-year developmental range of the instrument, rather than restricting the sample to a single age band, adds breadth to the validity evidence and extends the empirical reach of the short-form beyond the eight-to-twelve-year window examined in the original derivation study by Carl et al [7]. However, several limitations of the present investigation should be acknowledged transparently to appropriately bound the interpretations offered above. First, both constituent samples were drawn exclusively from the urban population of Tehran, limiting the generalizability of the findings to the broader Iranian population. Although Tehran is socioeconomically and ethnically diverse and includes families from many regions of the country, it cannot be assumed to be nationally representative. Consequently, the psychometric properties of the Persian short form should be confirmed in rural populations, other provinces, and communities with different socioeconomic and cultural characteristics before broader generalizations can be made. In addition, floor and ceiling effects were evaluated only for the overall sample and were not examined separately across demographic subgroups. Future studies should investigate these measurement properties by gender, age, and other relevant population characteristics to further evaluate the performance of the instrument. Second, the parental consent rate in the older cohort was relatively modest, raising the possibility of selection bias whereby the final sample may have overrepresented families with a higher degree of engagement with educational institutions or physical activity research. At the same time, the breadth of schools selected across socioeconomically diverse urban districts lends some confidence that the sample was not systematically skewed toward any single demographic subgroup. However, socioeconomic differences between the two constituent cohorts were not formally examined and therefore cannot be ruled out. Future studies should evaluate cohort comparability with respect to socioeconomic characteristics. In addition, the questionnaire was administered using different modes across the two cohorts,younger children completed the instrument individually with interviewer assistance, whereas older children completed it independently in classroom settings. Although interviewer assistance was necessary to ensure comprehension among younger children, these differences in administration mode may have introduced method effects independent of the underlying PL construct. Furthermore, interviewer presence may have influenced children's responses through social desirability or other interviewer-related effects, representing a potential source of measurement bias. Future studies should examine whether different administration modes influence the psychometric performance of the instrument. Third, the present study provides only partial psychometric validation of the Persian short form because several important measurement properties recommended by the COSMIN framework could not be evaluated using the available pooled datasets. In particular, criterion validity could not be examined because no objective or proxy measures of physical activity were available. This is an important limitation because the ability of the short form to predict physical activity was one of the key selection criteria used by Carl et al. [7] during its development. Consequently, it remains unknown whether the Persian version demonstrates criterion validity comparable to that of the original instrument. In addition, convergent and discriminant validity could not be assessed due to the absence of external measures of related and unrelated constructs, and the cross-sectional design precluded the evaluation of responsiveness and measurement error. Therefore, although the present findings support the instrument's structural validity, internal consistency, and measurement invariance, they should be interpreted as evidence of partial rather than comprehensive psychometric validation. Future longitudinal and multimethod studies should examine these remaining measurement properties. Fourth, the cross-sectional design of both constituent studies precludes any inference regarding the temporal stability of the Persian short form's psychometric properties,test–retest reliability data across an appropriate interval would be a valuable addition to the validity evidence base for this instrument. In addition, the Social domain consists of only two items, which likely attenuated its internal consistency and limits the precision of individual subscale scores. Future studies should examine whether adding or refining social-domain items can improve reliability while preserving the brevity of the short form. Finally, the psychometric evaluation of the Persian short form was based on responses extracted from the full 30-item PL-C Quest rather than on an independently administered 12-item questionnaire. Consequently, the observed psychometric properties may have been influenced by context or item-order effects associated with the full instrument. Future studies should administer the Persian short form as a standalone questionnaire to confirm that the present findings generalize to its intended independent use.

## Conclusion

The findings of this study provide initial support for the use of the Persian short form of the PL-C Quest among Iranian children aged 4–12 years. Overall, the results suggest that the proposed measurement model performs reasonably well in this population, with evidence supporting its factorial structure and generally acceptable internal consistency. The observed measurement invariance across gender and age groups, together with the stability of the factor structure in split-sample analyses, indicates that the questionnaire functions in a broadly consistent manner across these groups. Nevertheless, these findings should be interpreted with appropriate caution. Although the overall pattern of results is encouraging, the evidence for construct validity was not uniformly strong across all domains, indicating that further validation is warranted. As psychometric validation is an ongoing process, additional studies involving different populations and complementary forms of validity and reliability are needed to establish a more comprehensive evaluation of the instrument. Within these considerations, the Persian short form of the PL-C Quest may offer a practical and time-efficient option for assessing perceived PL in research and educational settings. However, its application should be viewed in light of the current evidence, which represents an important step in the validation process rather than definitive confirmation of the instrument's psychometric adequacy.

## Supplementary Information

Supplementary Material 1

Supplementary Material 2

## Data Availability

The datasets generated and analyzed during the current study are not publicly available due to participant confidentiality, but they are available from the corresponding author on reasonable request.

## Abbreviations

PL: Physical Literacy
APLF: Australian Physical Literacy framework
PL-C Quest: Physical Literacy in Children Questionnaire
AVE: Average Variance Extracted
CFA: Confirmatory Factor Analysis
HTMT: Heterotrait–Monotrait Ratio:
TLI: Tucker–Lewis Index
RMSEA: Root Mean Square Error of Approximation
SRMR: Standardized Root Mean Square Residual
