# Supplementary material for: Psychometric validation of the persian short form of the physical literacy in children questionnaire in Iranian children aged 4–12 years: factorial validity, internal consistency, and gender-based measurement invariance
Source: BMC Pediatr. 2026 Aug 3;26:1092. doi: 10.1186/s12887-026-07439-5 (PMC13726141; doi:10.1186/s12887-026-07439-5)
Supplement: Supplementary file 1 [file 12887_2026_7439_MOESM1_ESM.docx]

PHYSICAL LITERACY IN CHILDREN QUESTIONNAIRE: SCORING SHEET ID:

Grade/class:

Age:

Date:

Child’s name:_

School/Club:_

Date of birth:

Gender (circle): Male / Female / Non-binary / Prefer not to say

|  | Physical domain: How good are you at...? | | |  |
| --- | --- | --- | --- | --- |
| A LOT like me A BIT like me | Remember to check only one of the four boxes. | | | A LOT like me A BIT like me |
| ○ ○ | Some kids are very good at jumping | But | Other kids are not as good at jumping. | ○ ○ |
| ○ ○ | Some kids are very good at skateboarding | But | Other kids are not as good at skateboarding | ○ ○ |
| ○ ○ | Some kids are very good at throwing from the side | But | Other kids are not as good at throwing | ○ ○ |
| ○ ○ | Some kids are very good at running for a long time without getting tired | But | Other kids are not as good at running for a long time without getting tired | ○ ○ |
| ○ ○ | Some kids are very good at hanging for a long time without letting go | But | Other kids are not as good at hanging for a long time without letting go | ○ ○ |
| ○ ○ | Some kids are very good at doing a lot of jumps with a rope without their feet getting caught | But | Other kids are not as good at doing a lot of jumps with a rope and their feet get caught | ○ ○ |
| ○ ○ | Some children are very good at maintaining balance on a rock without shaking | But | Other children are not as good at maintaining balance on a rock and start to shake. | ○ ○ |
| ○ ○ | Some children are very good at touching their toes without bending their knees | But | Other children are not good at touching their toes and bend their knees. | ○ ○ |
| ○ ○ | Some children are very good at weaving around other kids while playing | But | Other children are not good at weaving around other kids while playing. | ○ ○ |
| ○ ○ | Some children are very good when strong muscles are needed, such as lifting a heavy stone | But | Other children are not good when strong muscles are needed, such as lifting a heavy stone. | ○ ○ |
| ○ ○ | Some children are very good at running forward as soon as they hear the starting sound | But | Other children are not good at running forward as soon as they hear the starting sound. | ○ ○ |
| ○ ○ | Some children are very good at running forward as soon as they hear the starting sound | But | Other children are not good at running forward as soon as they hear the starting sound. | ○ ○ |

|  | **Psychological domain: How do you feel about...?** | | |  |
| --- | --- | --- | --- | --- |
| A LOT like me A BIT like me | Remember to check only one of the four boxes. | | | A LOT like me A BIT like me |
| ○ ○ | Some children feel that they can be active and exercise whenever they want, and they have many reasons for doing so. | But | Other children do not find any good reasons to be active and exercise. | ○ ○ |
| ○ ○ | Some children feel that they can control their disappointment when they do not reach their goals. | But | Other children do not feel that they can control their disappointment when they miss the target. | ○ ○ |
| ○ ○ | Some children feel that they can adjust their speed to climb to the top of a hill. | But | Other children do not feel that they can adjust their speed to climb to the top of a hill. | ○ ○ |
| ○ ○ | Some children feel that they have a good perspective on their abilities. | But | Other children sometimes think they perform better than they actually do. | ○ ○ |
| ○ ○ | Some children feel that they have the confidence to try new active things, like sledding. | But | Other children do not feel that they have the confidence to try new active things, like sledding. | ○ ○ |
| ○ ○ | Some children feel that they enjoy being active in different ways because they find it fun. | But | Other children do not feel that they enjoy being active in different ways because they do not find it fun. | ○ ○ |
| ○ ○ | Some children feel that they have favorite places to gather and play. | But | Other children do not feel that they have favorite places to gather and play. | ○ ○ |

| **Social domain: Do you want to...?** | | | | |
| --- | --- | --- | --- | --- |
| A LOT like me A BIT like me | Remember to check only one of the four boxes. | | | A LOT like me A BIT like me |
| ○ ○ | Some kids want to shake hands with the other team after losing a game | But | Other kids do not want to shake hands with the other team after losing a game. | ○ ○ |
| ○ ○ | Some kids want to invite other kids to play with them | But | Other kids do not want to invite other kids to play with them. | ○ ○ |
| ○ ○ | Some kids want to participate in an activity or game where they can work together | But | Other kids do not want to participate in an activity or game where they can work together. | ○ ○ |
| ○ ○ | Some kids want to learn about the activities and games of other places and people | But | Other kids do not want to learn about the activities and games of other places and people. | ○ ○ |

| Cognitive domain: How do you think about...? Cognitive domain: How do you think about...? | | | | |
| --- | --- | --- | --- | --- |
| A LOT like me A BIT like me | Remember to check only one of the four boxes. | | | A LOT like me A BIT like me |
| ○ ○ | Some children think they can climb/cycle/ride and realize what might be in their way. | But | Other children don’t think they can cycle/ride but can and realize what might be in their way. | ○ ○ |
| ○ ○ | Some children think about the many reasons why physical activity is good for you. | But | Other children don’t think about the many reasons why physical activity is good for you. | ○ ○ |
| ○ ○ | Some children think about following the rules - like not jumping in the pool. | But | Other children don’t think about following the rules - and jump in the pool. | ○ ○ |
| ○ ○ | Some children think about another physical activity if their favorite one isn’t available. | But | Other children don’t think about another physical activity if their favorite one isn’t available. | ○ ○ |
| ○ ○ | Some children think about which path is best when they climb. | But | Other children don’t think about which path is best when they climb and they get stuck along the way. | ○ ○ |
| ○ ○ | Some children think about how to be in the right place to receive a pass. | But | Other children don’t think about how to be in the right place to receive a pass. | ○ ○ |
| ○ ○ | Some children think about where it is safe to swim  before they go into the water. | But | Other children don’t think about where it is safe to swim before they go into the water. | ○ ○ |
