# Supplementary material for: Psychometric validation of the persian short form of the physical literacy in children questionnaire in Iranian children aged 4–12 years: factorial validity, internal consistency, and gender-based measurement invariance
Source: BMC Pediatr. 2026 Aug 3;26:1092. doi: 10.1186/s12887-026-07439-5 (PMC13726141; doi:10.1186/s12887-026-07439-5)
Supplement: Supplementary file 2 [file 12887_2026_7439_MOESM2_ESM.docx]

**Supplementary File 1**

**ضمیمه – نسخه کوتاه شده پرسشنامه سواد بدنی در کودکان**

**شناسه:**

**درجه / کلاس:**

**سن:**

**تاریخ:**

**نام کودک:**

**مدرسه/باشگاه:**

**تاریخ تولد:**

**جنسیت (دایره بکشید): مذکر / مونث / غیر باینری / ترجیح می دهند نگویند**

| سواد بدنی در کودکان | | | | | |
| --- | --- | --- | --- | --- | --- |
| خیلی شبیه به من کمی شبیه به من | به یاد داشته باشید که فقط یکی از چهار کادر را علامت بزنید | | | خیلی شبیه به من کمی شبیه به من |  |
| ○ ○ | بعضی دیگر از بچه‌ها در پرش آن‌طور که باید خوب نیستند | اما | بعضی بچه‌ها در پرش خیلی خوب هستند | ○ ○ | 1 |
| ○ ○ | بعضی دیگر از بچه‌ها در دویدن برای مدت طولانی بدون خستگی آن‌طور که باید خوب نیستند. | اما | بعضی بچه‌ها در دویدن برای مدت طولانی بدون خستگی خیلی خوب هستند | ○ ○ | 02 |
| ○ ○ | بعضی دیگر از بچه‌ها در آویزان شدن برای مدت طولانی بدون رها کردن آن‌طور که باید خوب نیستند. | اما | بعضی بچه‌ها در آویزان شدن برای مدت طولانی بدون رها کردن خیلی خوب هستند | ○ ○ | 03 |
|  | بعضی دیگر از کودکان در دویدن بسیار سریع خوب نیستند. |  | برخی از کودکان در دویدن بسیار سریع خیلی خوب هستند |  | 04 |
| ○ ○ | برخی دیگر از کودکان دلیل خوبی برای فعال بودن و ورزش کردن نمی‌یابند | اما | برخی از کودکان احساس می‌کنند که هر زمان می‌توانند فعال باشند و ورزش کنند، دلایل زیادی برای این کار دارند | ○ ○ | 05 |
| ○ ○ | برخی دیگر از کودکان احساس نمی‌کنند که برای تجربه کردن کارهای جدید فعال، مانند سورتمه‌سواری، اعتماد به نفس دارند | اما | برخی از کودکان احساس می‌کنند که برای تجربه کردن کارهای جدید فعال، مانند سورتمه‌سواری، اعتماد به نفس دارند | ○ ○ | 06 |
| ○ ○ | برخی دیگر از کودکان احساس نمی‌کنند که دوست دارند به شیوه‌های مختلف فعال باشند، زیرا از آن لذت نمی‌برند | اما | برخی از کودکان احساس می‌کنند که دوست دارند به شیوه‌های مختلف فعال باشند، زیرا از آن لذت می‌برند | ○ ○ | 07 |
| ○ ○ | بعضی دیگر از بچه‌ها نمی‌خواهند بچه‌های دیگر را دعوت کنند تا با آن‌ها بازی کنند. | اما | بعضی از بچه‌ها می‌خواهند بچه‌های دیگر را دعوت کنند تا با آن‌ها بازی کنند | ○ ○ | 08 |
| ○ ○ | بعضی دیگر از بچه‌ها نمی‌خواهند درباره‌ی فعالیت‌ها و بازی‌های دیگر نقاط و مردم یاد بگیرند. |  | بعضی از بچه‌ها می‌خواهند درباره‌ی فعالیت‌ها و بازی‌های دیگر نقاط و مردم یاد بگیرند | ○ ○ | 09 |
| ○ ○ | برخی دیگر از کودکان به دلایل زیادی فکر نمی‌کنند که چرا فعالیت بدنی برای شما خوب است. | اما | برخی از کودکان به دلایل زیادی فکر می‌کنند که چرا فعالیت بدنی برای شما خوب است. | ○ ○ | 10 |
| ○ ○ | برخی دیگر از کودکان به این فکر نمی‌کنند که کدام مسیر بهترین است وقتی که بالا می‌روند و در راه می‌مانند. | اما | برخی از کودکان به این فکر می‌کنند که کدام مسیر بهترین است وقتی که بالا می‌روند. | ○ ○ | 11 |
| ○ ○ | برخی دیگر از کودکان به این فکر نمی‌کنند که چگونه در مکان مناسب باشند تا توپ به آنها پاس داده شود. | اما | برخی از کودکان به این فکر می‌کنند که چگونه در مکان مناسب باشند تا توپ به آنها پاس داده شود. | ○ ○ | 12 |
